# Double remission of chronic lymphocytic leukemia and secondary acute myeloid leukemia after venetoclax monotherapy: A case report

**DOI:** 10.1097/MD.0000000000024703

**Published:** 2021-02-12

**Authors:** Lei Wang, Na Lin

**Affiliations:** Department of Hematology, The First Affiliated Hospital of China Medical University, Shenyang, Liaoning Province, China.

**Keywords:** acute myeloid leukemia, chronic lymphocytic leukemia, complete remission, venetoclax

## Abstract

**Rationale:**

The abnormal expression of B-cell lymphoma-2 (Bcl-2) family members is often associated with the progression of the disease. Bcl-2 inhibitors (eg, venetoclax) were first reported to inhibit the proliferation of malignant lymphocytes and have a significant effect on patients with chronic lymphoblastic leukemia, but research on myeloid tumors is relatively delayed. Venetoclax was approved in 2018 for the treatment of acute myeloid leukemia (AML) patients who were not suitable for high-dose chemotherapy. The approval of venetoclax is an advance in the treatment of hematological tumors.

**Patient concerns:**

Here we report a 64-year-old male with an increased white blood cell (WBC) count (39.0 × 10^9^/L) and lymphocyte count (30.6 × 10^9^/L) on physical examination in July 2014. The patients were diagnosed with chronic lymphocytic leukemia (CLL) through bone marrow (BM) smears and immunophenotyping without any cytogenetic or molecular abnormalities. Chlorambucil was prescribed, WBC was stable between 15 × 10^9^/L and 25 × 10^9^/L in the past 6 years. He came to the hospital again in May 2020 and complained of fatigue for 2 weeks. WBC (16.7 × 10^9^/L) and lymphocyte (14.76 × 10^9^/L) counts were increased, hemoglobin (HGB) and platelet (PLT) were decreased in peripheral blood, which indicated the progression of the disease.

**Diagnoses:**

The patient was diagnosed as secondary AML after CLL based on the clinical and laboratory findings.

**Interventions:**

He achieved a morphological complete remission in both AML and CLL without any adverse reactions after one course of venetoclax monotherapy.

**Outcomes:**

He received standard daunorubicin and cytarabine combined with venetoclax as consolidation therapy and is now ready for allogeneic-hematopoietic stem cell transplantation.

**Lessons:**

Our case presents a challenge to traditional treatment. New drugs such as venetoclax have shown outstanding effects in this respect. High expression of Bcl-2 can identify the responders of venetoclax. These findings should be validated in future clinical trials. We fully believe that in the near future, the comprehensive use of targeted drugs with different mechanisms will not only improve the quality of life of patients, but also completely change the prognosis of patients with recurrent and refractory hematological malignancies.

## 1 Introduction

B-cell lymphoma-2 (Bcl-2) family proteins are involved in the pathogenesis of a variety of tumors, such as leukemia, breast cancer, and lymphoma. The abnormal expression of Bcl-2 family members is often associated with the progression of the disease. Studies have proven that Bcl-2 family members are involved in the chemotherapy resistance of various tumors and are closely related to the therapeutic effect of tumors.^[1,2]^ Bcl-2 inhibitors (venetoclax) were first reported to inhibit the proliferation of malignant lymphocytes and have a significant effect on patients with chronic lymphoblastic leukemia,^[3–6]^ Bcl-2 inhibitors have also been reported in multiple myeloma,^[7,8]^ but research on myeloid tumors is relatively delayed.^[9–13]^ Venetoclax selectively binds to Bcl-2,^[14]^ and the main effect of venetoclax is to prevent Bcl-2 from protecting cancer cells so that the cancer cells can undergo apoptosis similar to normal cells. Venetoclax was approved for the treatment of chronic lymphocytic leukemia (CLL)/small lymphocyte lymphoma, venetoclax combined with azacytidine, decitabine or low-dose cytarabine was approved in the treatment of adult acute myeloid leukemia (AML) in 2016 by the US FDA. Venetoclax was approved in 2018 for the treatment of AML patients who were not suitable for high-dose chemotherapy. The approval of venetoclax is an advancement in traditional medicine methods for blood tumors. The efficacy of the Bcl-2 inhibitor combined with azacytidine in AML is better than that of conventional chemotherapy.^[15]^ The incidence of AML after CLL is very rare. The outcome of this kind of patients is rather depressing, because the treatment for such patients is very limited, and only a few patients are suitable for intensive chemotherapy or allogeneic hematopoietic stem cell transplantation (allo-HSCT).^[16]^ With the development of new diagnostic methods and innovative therapies, the treatment mode of patients is gradually improved. Here, we report a 64-year-old patient diagnosed with AML after CLL. He achieved a morphological complete remission (CR) in both AML and CLL without any adverse reactions after one course of venetoclax monotherapy. Then, he received standard daunorubicin and cytarabine combined with venetoclax as consolidation therapy and is now ready for allo-HSCT.

## 2 Case report

**Figure 1:**
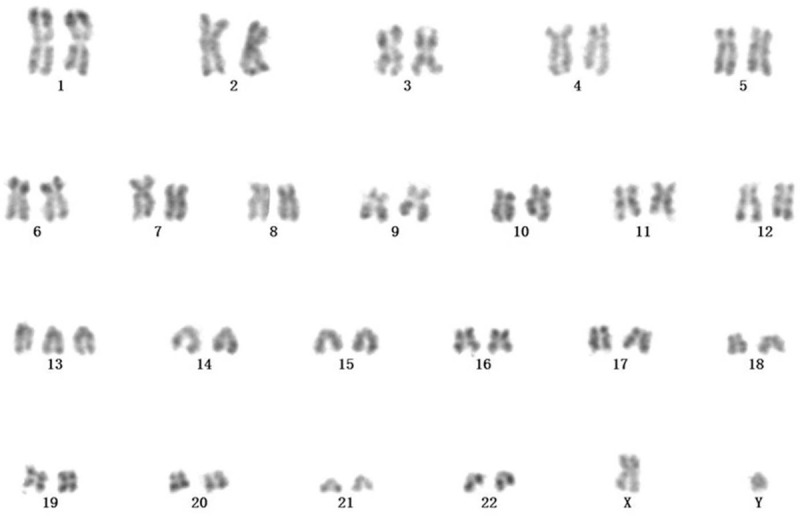
Karyotype analysis showed 47, XY,+ 13[5]/48,idem,+ 13[2]/46,XY[13].

**Figure 2:**
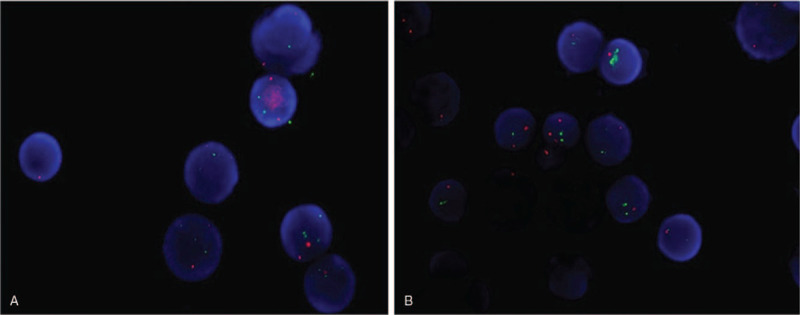
Fluorescent in situ hybridization was done on bone marrow samples revealed the abnormal on 13q14 and 13q14.3. A: Abnormal on 13q14, B: Abnormal on 13q14.3.

We report a 64-year-old male with an increased white blood cell (WBC) count (39.0 × 10^9^/L) and lymphocyte count (30.6 × 10^9^/L) on physical examination in July 2014. The patients were diagnosed with CLL through BM smears and immunophenotyping (abnormal lymphocyte ratio 47.52%) without any cytogenetic or molecular abnormalities. Chlorambucil was prescribed, WBC was stable between 15 × 10^9^/L and 25 × 10^9^/L in the past 6 years. He came to the hospital again in May 2020 and complained of fatigue for 2 weeks. WBC (16.7 × 10^9^/L) and lymphocyte (14.76 × 10^9^/L) counts were increased, HGB and PLT were decreased in peripheral blood, which indicated the progression of the disease. Physical and chemical examination were unremarkable except for a single lesion of the pulmonary parenchyma (4.4 × 3.5 cm) and slight splenomegaly. Neither superficial nor deep lymph node enlargement was found. He was diagnosed with secondary AML concomitant with CLL according to the BM smears, BM biopsy and immunophenotyping (abnormal monoclonal B lymphocyte ratio 46.6% and malignant myeloid immature cells ratio 27.1%) without any molecular abnormalities other than the presence of DNMT3A, NOTCH1, CSF3R, SRSF2, and SF3B1 mutations. Karyotype analysis showed 47,XY,+ 13[5]/48,idem,+ 13[2]/46,XY[13] (Fig. 1), and the hot spot fusion gene of leukemia was negative. Fluorescent in situ hybridization was done on bone marrow samples revealed the abnormal on 13q14 and 13q14.3 (Fig. 2). Because of the extremely low PLT, he could not undergo lung biopsy. Bcl-2 was positive in both peripheral blood and bone marrow by immunohistochemistry. So a single dose of venetoclax (400 mg once a day, according to the literature^[16]^) was prescribed from June 2020, combined with measures to prevent tumor lysis syndrome. A normalization of the peripheral blood count was observed, along with clearance of the lymphocytosis. He achieved a morphological CR with a percentage of clonal small lymphocytes of 2.45% and a percentage of malignant myeloid immature cells of less than 1% by immunophenotyping. Physical examination revealed no reduction in the pulmonary parenchyma mass. Subsequently, he received standard daunorubicin and cytarabine (daunorubicin 45 mg/m^2^, cytarabine 100 mg/m^2^) combined with venetoclax as a consolidation treatment and achieved a molecular complete remission without any monoclonal B lymphocytes or malignant myeloid immature cells by immunophenotyping. Bone marrow suppression appeared quickly and lasted for two months, which may be associated with the long history of CLL. He underwent open lung surgery after his PLT returned to normal. The pulmonary mass was resected, flow cytometry showed that there were no malignant clones suggested CLL or AML, but 13q14 and 13q22 were found by fluorescence in situ hybridization, lung biopsy revealed inflammatory pseudotumor. We speculated that the 13q detected by fish was due to mixing of peripheral blood. The patient is now ready for allo-HSCT.

## 3 Discussion

With the progress of medicine and the prolongation of the human life span, the proportion of patients with 2 tumors has increased significantly. However, patients with multiple tumors were often excluded from clinical trials, therefore, the clinical characteristics, treatment and prognosis of these patients are rarely reported. To the best of our knowledge, this is the first case of patients diagnosed with AML after CLL treated with venetoclax monotherapy. Venetoclax can highly selectively bind to Bcl-2 and induce the apoptosis. In 2014, a phase 2 clinical trial of venetoclax in relapsed/refractory AML with a total of 32 patients enrolled was launched, 19% relapsed and refractory AML patients got complete response/complete response with incomplete blood recovery (CR/CRi) with venetoclax.^[17]^ Pasquale Niscola reported that a 75-year-old woman developed CLL and AML 10 years after the diagnosis of MDS-RAEB2,^[16]^ venetoclax alone achieved transient CR, but the patient ultimately died. According to the results of an international phase Ib/II study, more than half of elderly patients with AML achieved CR rapidly after treatment with venetoclax and low-dose cytarabine.^[18]^ As we speculated, venetoclax has a therapeutic effect on both CLL and AML malignant clones. As a single oral drug, the novel agent venetoclax showed very good effects in these patients. However, venetoclax also has disadvantages. It has been reported that patients treated with venetoclax have a relatively short remission and relapse soon, drug resistance to the original chemotherapeutic drugs is an urgent problem to be solved.^[19]^ Therefore, patients should receive HSCT as soon as possible to obtain a sustained CR.^[20]^ However, there are also reports that CLL and subsequent AML were diagnosed in patients without any treatment, showing 2 completely different and unrelated malignant tumors. A report showed that a 70-year-old man was diagnosed with CLL and had never been treated, he was diagnosed with AML five years later, this confirms our conjecture.^[21]^ The occurrence of CLL and AML suggested that they may have the same tumor stem cells. Venetoclax monotherapy can simultaneously achieve CR in AML and CLL, which is sufficient to demonstrate the unique efficacy of venetoclax in these patients. Venetoclax as a single drug in the treatment of relapsed/refractory AML, the effective rate was 19%.^[17]^ However, there is no report about venetoclax monotherapy in newly diagnosed AML. At present, the therapeutic effect of lymphoproliferative diseases associated with AML is still poor. Chemotherapy free induction like venetoclax monotherapy without serious toxic effects could improve the life quality of the patients and reduce the mortality rate related to chemotherapy. High expression of Bcl-2 can identify the responders of venetoclax. These findings should be validated in future clinical trials.

Despite the frustrating situation, patients are getting more accurate diagnosis and individualized treatment with the application of new diagnostic methods and innovative therapies. We fully believe that in the near future, the comprehensive use of targeted drugs with different mechanisms will not only improve the quality of life of patients, but also completely change the prognosis of patients with recurrent and refractory hematological malignancies.

## Acknowledgments

The authors thank American Journal Experts who provided language editing.

## Author contributions

**Data curation:** Lei Wang.

**Formal analysis:** Lei Wang.

**Funding acquisition:** Lei Wang.

**Investigation:** Lei Wang.

**Software:** Lei Wang.

**Writing – original draft:** Lei Wang.

**Writing – review & editing:** Na Lin.

## Abbreviations

Abbreviations: AML = acute myeloid leukemia, Bcl-2 = B-cell lymphoma-2, CLL = chronic lymphocytic leukemia, CR = complete remission, HSCT = hematopoietic stem cell transplantation, PLT = platelet count, WBC = white blood cell.
